# Restoring prearthritic alignment improves joint perception in medial unicompartmental knee arthroplasty

**DOI:** 10.1002/jeo2.70389

**Published:** 2025-08-05

**Authors:** Zhaolun Wang, Wang Deng, Yixin Zhou, Yong Huang, Shaoyi Guo, Yunfeng Zhang

**Affiliations:** ^1^ Department of Orthopaedic Surgery, Beijing Jishuitan Hospital Capital Medical University Beijing China; ^2^ Fourth Clinical College of Peking University Beijing China

**Keywords:** arithmetic hip‐knee‐ankle angle, forgotten joint, joint perception, prearthritic alignment, retrospective cohort study, unicompartmental knee arthroplasty

## Abstract

**Purpose:**

This study aims to investigate whether restoring prearthritic alignment improves joint perception in medial unicompartmental knee arthroplasty (UKA).

**Methods:**

This retrospective cohort study analysed 244 patients who underwent nonrobotic‐assisted medial fixed‐bearing UKA between 2015 and 2018 with a minimum 2‐year follow‐up. Patients were categorised into prearthritic and nonprearthritic alignment groups based on the difference between their postoperative alignment and prearthritic hip‐knee‐ankle angle. Postoperative outcomes, including the Forgotten Joint Score (FJS‐12), Oxford Knee Score (OKS) and Western Ontario and McMaster Universities Osteoarthritis Index (WOMAC), Knee Injury and Osteoarthritis Outcome Score (KOOS), Oxford Knee Score (OKS) and University of California Los Angeles (UCLA) activity score were compared between the groups. Additional analysis was performed in a subgroup of patients with constitutional varus alignment (CPAK types I, IV and VII). Multivariable logistic regression was used to identify predictors of achieving a forgotten joint.

**Results:**

Patients with restored prearthritic alignment had significantly higher FJS‐12 (71.9 vs. 63.4, *p* = 0.005), OKS (40.7 vs. 38.1, *p* = 0.003) and WOMAC (91.2 vs. 88.1, *p* = 0.017) scores compared to those with nonprearthritic alignment. In the constitutional varus subgroup, prearthritic alignment was associated with higher FJS‐12, UCLA, OKS and KOOS ADL scores. The prearthritic alignment group also had a higher likelihood of achieving the ‘forgotten joint’ state. A 1‐degree deviation from prearthritic alignment was associated with a 21% decrease in the probability of achieving a forgotten joint.

**Conclusion:**

Restoring prearthritic alignment in UKA is associated with improved postoperative joint perception and function, especially in patients with constitutional varus alignment. This personalised alignment approach may lead to better outcomes than the traditional goal of neutral alignment. Further research with a longer follow‐up period is required to validate these findings and explore their impact on prosthesis survival.

**Levels of Evidence:**

Level II.

**Abbreviations**
aHKA: arithmetic hip‐knee‐ankle angle
CCI: Charlson comorbidity index
CPAK: coronal plane alignment of the knee
FJS‐12: Forgotten Joint Score
JLO: joint line obliquity
KOOS: Knee Injury and Osteoarthritis Outcome Score
mHKA: mechanical hip‐knee‐ankle angle
mLDFA: mechanical lateral distal femoral angle
MPTA: medial proximal tibial angle
OA: osteoarthritis
OKS: Oxford Knee Score
PASS: patient acceptable symptom state
UCLA: University of California Los Angeles activity score
UKA: unicompartmental knee arthroplasty
WOMAC: Western Ontario and McMaster Universities Osteoarthritis Index

## INTRODUCTION

Unicompartmental knee arthroplasty (UKA) is an effective alternative to total knee arthroplasty (TKA) for treating anteromedial osteoarthritis (OA) of the knee. UKA offers advantages, such as reduced blood loss, faster postoperative recovery and improved postoperative joint perception [8, 17, 19]. Previous studies have reported the importance of postoperative alignment in the success of UKA [15, 24, 26, 28]. However, the optimal postoperative alignment for UKA remains controversial. Traditionally, neutral alignment is believed to ensure postoperative biomechanical balance and prosthesis longevity [12, 24, 33]. Conversely, recent studies suggested that maintaining a slight varus alignment (1°–4°) yields better postoperative function for medial UKA [26, 32, 35]. However, a fixed target value may overlook individual differences. A recent study introduced the coronal plane alignment of the knee (CPAK) classification to guide personalised postoperative alignment and proposed the concept of arithmetic hip‐knee‐ankle angle (aHKA), which may better represent patients' constitutional limb alignment and improve soft‐tissue tensioning [21]. Therefore, some authors advocate for personalised UKA alignment by measuring the aHKA to restore a patient's prearthritic alignment [11]. However, high‐quality evidence supporting the use of prearthritic alignment in UKA is lacking.

The ‘forgotten joint’ is believed to be the ultimate goal of joint arthroplasty [3]. The Forgotten Joint Score (FJS‐12), based on this concept, has a low ceiling effect [9], making it a better tool for distinguishing patients receiving UKA [31]. Therefore, this study aimed to investigate the effect of restoring aHKA on achieving the ‘forgotten joint’ state after fixed‐bearing medial UKA.

## MATERIALS AND METHODS

### Patient selection

This study included patients who underwent medial fixed‐bearing UKA (ZUK, Zimmer) between May 2015 and December 2018. These patients were all cemented and manual instrumentation was used. For these cases, we targeted a neutral or mild varus (<5°) alignment, with adjustments made within this range to obtain optimal soft tissue tension. Patients were considered as candidates for UKA based on these criteria: (1) isolated anteromedial knee OA; (2) preoperative varus deformity ≤15° and correctible under valgus stress; (3) fixed flexion deformity ≤10°; (4) preoperative range of motion ≥90°; and (5) intact and functional cruciate ligaments. Patients were excluded for this study if they could not complete the FJS‐12 due to medical comorbidities, had inadequate preoperative or postoperative radiographs, or failed to complete the minimum 2‐year follow‐up. Consequently, 244 patients who underwent UKA met the inclusion criteria. The median follow‐up period was 3.5 years.

### Radiographic evaluation

**Figure 1:**
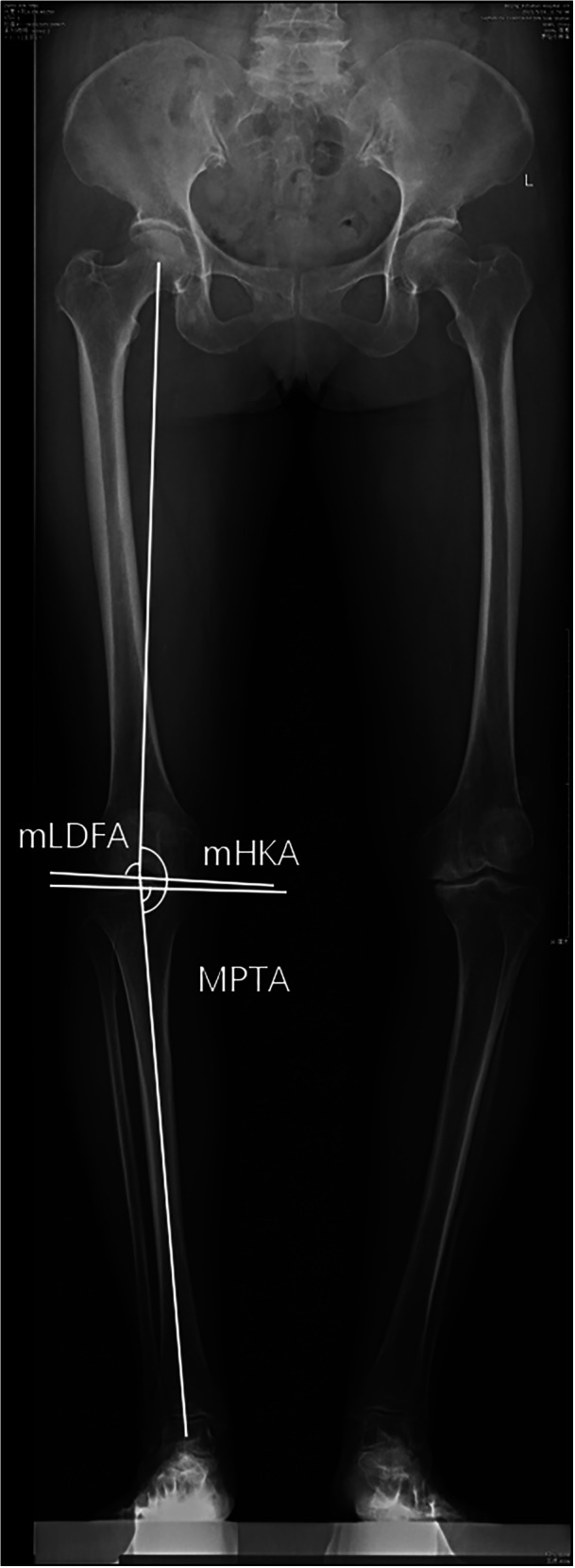
Measurements of mechanical hip‐knee‐ankle angle (mHKA), mechanical lateral distal femoral angle (mLDFA) and medial proximal tibial angle (MPTA).

The following parameters were assessed on preoperative long‐standing anteroposterior radiographs (Figure 1): (1) mechanical HKA (mHKA), the angle formed by the femoral and tibial mechanical axes; [22] (2) mechanical lateral distal femoral angle (mLDFA), the lateral angle between the femoral mechanical axis and the distal femoral joint line; [22] and (3) medial proximal tibial angle (MPTA), the medial angle between the tibial mechanical axis and proximal tibial joint line. The aHKA was calculated using the formula: aHKA = MPTA − LDFA, with negative values indicating varus alignment and positive values indicating valgus alignment. Joint line obliquity (JLO) was calculated as follows: JLO = MPTA + LDFA. Patients were classified into different CPAK subtypes based on their preoperative aHKA and JLO [21]. The postoperative mHKA was assessed using long‐standing anteroposterior radiographs. The difference between postoperative HKA and aHKA (ΔaHKA) was calculated to assess the restoration from prearthritic alignment. The difference between postoperative HKA and neutral alignment (ΔmHKA) was also calculated. Patients were categorised into prearthritic (ΔaHKA ≤ 2°)/nonprearthritic (ΔaHKA > 2°) and neutral (ΔmHKA ≤ 2°)/nonneutral (ΔmHKA > 2°) groups. Measurements were performed independently by one researcher. Repeated measurements were performed by another independent researcher on a sample of 100 randomly selected cases for the calculation of intraclass correlation coefficient (ICC), which can be found in Table S1.

### Patient follow‐up

Postoperative follow‐ups were conducted at 3, 6 and 12 months, and annually thereafter. At the most recent follow‐up, the patients completed the FJS‐12, Western Ontario and McMaster Universities Osteoarthritis Index (WOMAC), Knee Injury and Osteoarthritis Outcome Score (KOOS), Oxford Knee Score (OKS) and University of California Los Angeles (UCLA) activity score. All of these scores have been translated and validated in the patients' native language [5, 6, 7, 18, 27]. Postoperative complications, including any reoperations and deep infections, were recorded. The primary outcome was the achievement of a forgotten joint state assessed by FJS‐12 [31], while the secondary outcome was the percentage of patients who reached the patient acceptable symptom state (PASS) for FJS‐12 and WOMAC scores [31].

### Data analysis

Statistical analysis was performed using R software (version 3.3.1). Shapiro–Wilk tests were used to assess normality. Normally distributed continuous variables were expressed as means and standard deviations and compared between groups using an independent *t*‐test. Nonnormally distributed continuous variables were expressed as medians and quartiles and compared between groups using the Mann–Whitney *U* test. Categorical variables were compared using the chi‐square and Fisher's exact test. Additionally, a multivariable logistic regression model was used to explore the effects of ΔaHKA and ΔmHKA on the primary outcome (achievement of forgotten joint), adjusting for variables including age, body mass index (BMI), sex, Charlson comorbidity index (CCI), and preoperative aHKA, mHKA and JLO. The sample size calculation was based on our prior follow‐up results: the difference of FJS‐12 between patients in the prearthritis alignment group and the control group was estimated to be 8.0 with a standard deviation of 20.0. Assuming an equal number of patients in the prearthritis alignment and control groups, as well as alpha set at 0.05 and power set at 0.8, the final total number of patients required was 200.

## RESULTS

Overall, 50.8% of patients achieved prearthritic alignment, while only 33.3% achieved neutral alignment postoperatively. There were no significant differences between the prearthritic and nonprearthritic alignment groups in terms of age, sex, BMI and CCI (Table 1). Additionally, there was no significant difference between the two groups regarding preoperative and postoperative mHKA and CPAK subtypes. Conversely, the nonneutral alignment group exhibited more varus in the preoperative mHKA and aHKA compared to the neutral alignment group, and there were more patients with CPAK type I in the nonneutral alignment group (Table 2). There were no significant differences in age, sex, BMI and CCI between the neutral and nonneutral alignment groups.

**Table 1:** Patient demographics for the prearthritically aligned and nonprearthritically aligned groups.

	Prearthritically aligned	Nonprearthritically aligned	
	*n* = 124	*n* = 120	*p*
Age (years)^a^	61.9 ± 7.9	63.2 ± 8.5	0.237
BMI (kg/m^2^)^a^	27.0 ± 3.5	27.2 ± 3.4	0.651
CCI^b^	2 (1−3)	2 (1−3)	0.201
Preoperative mHKA (°)^a^	−6.2 ± 3.0	−6.9 ± 2.8	0.056
mLDFA (°)^a^	88.7 ± 2.1	88.9 ± 2.8	0.497
MPTA (°)^a^	85.7 ± 2.1	85.4 ± 2.4	0.294
aHKA (°)^a^	−3.0 ± 2.2	−3.5 ± 3.7	0.191
JLO (°)^a^	174.4 ± 3.5	174.3 ± 3.6	0.857
Postoperative mHKA (°)^a^	−2.9 ± 2.4	−3.0 ± 3.0	0.690
Sex^c^			0.148
Female	86 (69.4)	94 (78.3)	
Male	38 (30.6)	26 (21.7)	
CPAK^c^			0.367
I	64 (51.6)	63 (52.5)	
II	30 (24.2)	25 (20.8)	
III	1 (0.8)	6 (5.0)	
IV	20 (16.1)	17 (14.2)	
V	9 (7.3)	7 (5.8)	
VI	0 (0)	1 (0.8)	
VII	0 (0)	1 (0.8)	

Abbreviations: aHKA, arithmetic hip‐knee‐ankle angle; BMI, body mass index; CCI, Charlson Comorbidity Index; CPAK, coronal plane alignment of the knee; JLO, joint line obliquity; mHKA, mechanical hip‐knee‐ankle angle; mLDFA, mechanical lateral distal femoral angle; MPTA, medial proximal tibial angle.

^a^ Presented as mean ± standard deviation. The differences between groups were analysed using the Student's *t*‐test.

^b^ Presented as median (quartile). Differences between groups were analysed using the Mann–Whitney *U* test.

^c^ Presented as number (percentage). Differences between the groups were analysed using the chi‐square test or Fisher's exact test.

**Table 2:** Patient demographics for the neutrally aligned and nonneutrally aligned groups.

	Neutrally aligned	Nonneutrally aligned	
	*n* = 81	*n* = 163	*p*
Age (years)^a^	63.0 ± 8.0	62.3 ± 8.3	0.498
BMI (kg/m^2^)^a^	26.8 ± 2.9	27.2 ± 3.7	0.407
CCI^b^	2 (1−3)	2 (1−3)	0.937
Preoperative mHKA (°)^a^	−4.8 ± 2.4	−7.4 ± 2.7	<0.001
mLDFA (°)^a^	87.9 ± 2.5	89.3 ± 2.3	<0.001
MPTA (°)^a^	86.0 ± 2.2	85.4 ± 2.2	0.029
aHKA (°)^a^	−1.9 ± 2.7	−3.9 ± 3.0	<0.001
JLO (°)^a^	173.9 ± 3.8	174.6 ± 3.4	0.159
Postoperative mHKA (°)^a^	−0.6 ± 1.0	−4.1 ± 2.5	<0.001
Sex^c^			0.937
Female	59 (72.8)	121 (74.2)	
Male	22 (27.2)	42 (25.8)	
CPAK^c^			<0.001
I	29 (35.8)	98 (60.1)	
II	29 (35.8)	26 (16)	
III	5 (6.2)	2 (1.2)	
IV	10 (12.3)	27 (16.6)	
V	7 (8.6)	9 (5.5)	
VI	0 (0)	1 (0.6)	
VII	1 (1.2)	0 (0)	

Abbreviations: aHKA, arithmetic hip‐knee‐ankle angle; BMI, body mass index; CCI, Charlson Comorbidity Index; CPAK, coronal plane alignment of the knee; JLO, joint line obliquity; mHKA, mechanical hip‐knee‐ankle angle; mLDFA, mechanical lateral distal femoral angle; MPTA, medial proximal tibial angle.

^a^ Presented as mean ± standard deviation. The differences between groups were analysed using the student's *t*‐test.

^b^ Presented as median (quartile). Differences between groups were analysed using the Mann–Whitney *U* test.

^c^ Presented as number (percentage). Differences between the groups were analysed using the chi‐square test or Fisher's exact test.

The FJS‐12, OKS and WOMAC scores were significantly higher in the prearthritic alignment group compared to the nonprearthritic alignment group (Table 3). Additionally, the prearthritic alignment group had higher activities of daily living (ADL) and symptom scores on the KOOS scale. Although there was a trend towards higher UCLA scores in the prearthritic alignment group, the difference did not reach statistical significance. Additional analysis was performed in the subgroup of patients with constitutional varus alignment (CPAK types I, IV and VII), which showed that prearthritic alignment was associated with higher FJS‐12, UCLA, OKS and KOOS ADL scores (Table 4). There was no significant difference in postoperative PROMs between the neutral and nonneutral alignment groups (Table 5).

**Table 3:** Postoperative patient‐reported outcome measures for the prearthritically aligned and nonprearthritically aligned groups.

	Prearthritically aligned	Nonprearthritically aligned	
	*n* = 124	*n* = 120	*p*
FJS‐12^a^	71.9 ± 21.1	63.4 ± 25.5	0.005
UCLA^b^	4 (3–4)	4 (3–4)	0.077
KOOS symptoms^a^	89.4 ± 11.6	85.7 ± 13.9	0.028
KOOS pain^a^	91.8 ± 11.3	89.2 ± 13.4	0.111
KOOS ADL^a^	90.7 ± 9.6	87.2 ± 12.2	0.013
KOOS sport^a^	60.3 ± 24.3	55.7 ± 25.7	0.148
KOOS QoL^a^	74.2 ± 17.7	70.8 ± 20.4	0.171
WOMAC^a^	91.2 ± 9.4	88.1 ± 11.0	0.017
OKS^a^	40.7 ± 5.8	38.1 ± 7.5	0.003

Abbreviations: ADL, activities of daily living; KOOS, Knee Injury and Osteoarthritis Outcome Score; OKS; Oxford Knee Score; QoL, quality of life; UCLA, University of California Los Angeles; WOMAC, Western Ontario and McMaster Universities Osteoarthritis Index.

^a^ Presented as mean ± standard deviation. The differences between groups were analysed using the student's *t*‐test.

^b^ Presented as median (quartile). Differences between groups were analysed using the Mann–Whitney *U* test.

**Table 4:** Postoperative patient‐reported outcome measures in patients with constitutional varus alignment.

	Prearthritically aligned	Nonprearthritically aligned	
	*n* = 84	*n* = 81	*p*
FJS‐12^a^	73.8 ± 18.1	63.5 ± 24.7	0.003
UCLA^b^	4 (3.75–5)	4 (3–4)	0.033
KOOS symptoms^a^	89.3 ± 12.1	85.8 ± 13.5	0.080
KOOS pain^a^	91.5 ± 10.9	89.9 ± 12.5	0.358
KOOS ADL^a^	91.0 ± 8.7	87.6 ± 11.7	0.034
KOOS sport^a^	62.4 ± 22.4	56.2 ± 24.1	0.091
KOOS QoL^a^	74.7 ± 17.3	71.3 ± 21.3	0.263
WOMAC^a^	91.4 ± 8.6	88.5 ± 10.4	0.054
OKS^a^	41.1 ± 5.3	38.4 ± 7.0	0.006

Abbreviations: ADL, activities of daily living; KOOS, Knee Injury and Osteoarthritis Outcome Score; OKS; Oxford Knee Score; QoL, quality of life; UCLA, University of California Los Angeles; WOMAC, Western Ontario and McMaster Universities Osteoarthritis Index.

^a^ Presented as mean ± standard deviation. The differences between groups were analysed using the student's *t*‐test.

^b^ Presented as median (quartile). Differences between groups were analysed using the Mann–Whitney *U* test.

**Table 5:** Postoperative patient‐reported outcome measures for the neutrally aligned and nonneutrally aligned groups.

	Neutrally aligned	Nonneutrally aligned	
	*n* = 81	*n* = 163	*p*
FJS‐12^a^	66.1 ± 23.2	68.5 ± 24	0.447
UCLA^b^	4 (3–4)	4 (3–5)	0.268
KOOS symptoms^a^	87.3 ± 13.6	87.8 ± 12.6	0.784
KOOS pain^a^	89.8 ± 12	90.9 ± 12.6	0.522
KOOS ADL^a^	87.5 ± 12.5	89.7 ± 10.3	0.180
KOOS sport^a^	58.0 ± 24.7	58.1 ± 25.3	0.975
KOOS QoL^a^	71.2 ± 18.9	73.2 ± 19.2	0.444
WOMAC^a^	88.5 ± 11.2	90.3 ± 9.9	0.219
OKS^a^	38.4 ± 7.1	40.0 ± 6.6	0.083

Abbreviations: ADL, activities of daily living; KOOS, Knee Injury and Osteoarthritis Outcome Score; OKS; Oxford Knee Score; QoL, quality of life; UCLA, University of California Los Angeles; WOMAC, Western Ontario and McMaster Universities Osteoarthritis Index.

^a^ Presented as mean ± standard deviation. The differences between groups were analysed using the student's *t*‐test.

^b^ Presented as median (quartile). Differences between groups were analysed using the Mann–Whitney *U* test.

Patients with prearthritic alignment were more likely to reach the PASS thresholds for the FJS‐12 (91.1% vs. 79.2%, *p* = 0.014) and WOMAC (88.7% vs. 76.7%, *p* = 0.020) compared to patients with nonprearthritic alignment (Table 6). There was also a trend for more patients in the prearthritic alignment group to achieve forgotten joints (34.7% vs. 24.2%, *p* = 0.097). There was no significant difference in the proportion of patients meeting these thresholds between the neutral and nonneutral alignment groups.

**Table 6:** Proportion of patients meeting the thresholds for forgotten joint and patient satisfaction.

	Prearthritically aligned	Nonprearthritically aligned	*p*	Neutrally aligned	Nonneutrally aligned	*p*
	*n* = 124	*n* = 120		*n* = 81	*n* = 163	
Forgotten joint^a^	43 (34.7)	29 (24.2)	0.097	19 (23.5)	53 (32.5)	0.190
FJS‐12 PASS^a^	113 (91.1)	95 (79.2)	0.014	69 (85.2)	139 (85.3)	0.984
WOMAC PASS^a^	110 (88.7)	92 (76.7)	0.020	64 (79.0)	138 (84.7)	0.357

*Note*: Differences between the groups were analysed using the chi‐square test or Fisher's exact test. Abbreviations: PASS, patient acceptable symptom state; WOMAC, Western Ontario and McMaster Universities Osteoarthritis Index.

^a^ Presented as a number (percentage).

Finally, multivariable logistic regression analyses showed that the difference between postoperative HKA and aHKA (ΔaHKA) was an independent predictor of postoperative forgotten joint achievement (Table 7). A 1‐degree difference from the aHKA resulted in a 21% decrease in the probability of achieving postoperative forgotten joints. In contrast, ΔmHKA, preoperative aHKA, and preoperative JLO had no significant effect on the postoperative achievement of forgotten joint.

**Table 7:** Multivariable logistic regression analysis exploring the effect of ΔaHKA and ΔmHKA on the achievement of forgotten joint.

	OR (95% CI)	*p*
ΔaHKA	0.79 (0.66–0.95)	0.013
ΔmHKA	0.91 (0.76–1.09)	0.322
Age	0.94 (0.88–1.00)	0.060
BMI	1.01 (0.93–1.11)	0.743
Male sex	1.07 (0.55–2.05)	0.842
CCI	1.83 (1.15–2.94)	0.011
aHKA	0.96 (0.83–1.10)	0.528
JLO	1.07 (0.99–1.17)	0.093
Preoperative mHKA	1.06 (0.91–1.24)	0.470

Abbreviations: aHKA, arithmetic hip‐knee‐ankle angle; BMI, body mass index; CCI, Charlson Comorbidity Index; CI, confidence interval; JLO, joint line obliquity; mHKA, mechanical hip‐knee‐ankle angle; OR, odds ratio.

## DISCUSSION

The most important finding of this study was that targeting the patient's prearthritic alignment was associated with better postoperative joint perception, specifically with a higher probability of achieving the forgotten joint state. This effect was independent of preoperative factors such as age, sex, BMI, comorbidities, preoperative mHKA and aHKA. With each 1‐degree deviation from prearthritic alignment postoperatively, the probability of achieving a forgotten joint decreased by 21%.

Proper lower limb alignment is crucial for the success of UKA; [13] however, the optimal alignment after UKA is still controversial. Hernigou et al. studied 58 cases of medial fixed‐bearing UKA with an average 15‐year follow‐up, finding that overcorrecting the varus deformity was associated with a higher risk of degeneration in the lateral compartment, while residual varus deformity may lead to an increased risk of tibial polyethylene wear [12]. A retrospective study of 104 medial fixed‐bearing UKAs by Zuiderbaan et al. showed that patients with 1°–4° postoperative varus had the highest WOMAC scores [35]. Similarly, a study by Wang et al. involving 302 cases of medial fixed‐bearing UKA demonstrated that the highest probability of achieving a forgotten joint was observed in patients with a postoperative varus of 1.5°–4° [32]. Conversely, Vasso et al. studied 125 medial fixed‐bearing UKAs and found that patients with a postoperative varus of 5°–7° had the best postoperative functional results [29].

This inconsistency in the results of these studies suggests that there is no ‘one‐size‐fits‐all’ target alignment for patients receiving UKA. A study of 250 healthy young people showed that 32% of men and 17% of women had a constitutional varus of >3° [4]. For these individuals, restoring the neutral mechanical axis may be considered an overcorrection, potentially resulting in an unnatural knee sensation. Therefore, some authors advocate personalised UKA alignment by measuring the aHKA to restore the patient's prearthritic alignment [11, 16, 20, 21]. For example, Plancher et al. compared 127 prearthritically aligned (within 3° of the aHKA) versus 23 nonprearthritically aligned fixed‐bearing medial UKAs and found that patients with prearthritic alignment had longer prosthetic survival, and higher KOOS ADL and sports scores compared to outliers [23]. Bayoumi et al. analysed 537 medial fixed‐bearing UKAs and found that under‐correction from the prearthritic alignment was associated with lower Kujala scores [1]. Vossen et al. analysed 618 knees with a minimum 2‐year follow‐up after robotic‐assisted medial UKA and found that preservation of the CPAK phenotype and restoration of prearthritic coronal alignment were associated with significantly higher Kujala scores compared to altered phenotypes [30]. However, these studies only performed univariate analysis, and they did not perform subgroup analysis in patients with constitutional varus alignment. Additionally, the postoperative assessments in these studies did not include PROMs with low ceiling effects, such as the FJS‐12 and UCLA scores, which are better diagnostic tools for patients undergoing UKA.

This study findings revealed that prearthritic alignment was associated with higher FJS‐12, OKS, WOMAC, KOOS symptoms and ADL scores. In addition, in the constitutional varus subgroup, prearthritic alignment was associated with higher FJS‐12, UCLA, OKS and KOOS ADL scores. Furthermore, multivariable analysis confirmed that the difference between postoperative HKA and aHKA was an independent predictor of achieving a forgotten joint. The forgotten joint is considered the goal of joint arthroplasty, which is a highly functional state that may be associated with proper postoperative ligament tension [10, 14, 25]. Restoration of prearthritic alignment may enhance joint perception by optimising ligament tension.

Our multivariable regression analysis revealed that the preoperative aHKA and JLO had no significant effects on the achievement of forgotten joints. Furthermore, while the preoperative CPAK classification may influence the achievement of postoperative neutral alignment, it does not affect the achievement of prearthritic alignment. This finding contrasts with the results of Bayoumi et al., who reported that varus prearthritic alignment was associated with postoperative overcorrection relative to the aHKA [1]. This discrepancy may be related to the surgical technique and use of robot‐assisted surgery, as another study on nonrobotassisted UKA did not observe this difference [23]. In addition, it has been reported in previous study that both robotically and nonrobotically assisted, fixed‐bearing medial UKA can effectively restore prearthritic coronal alignment [2, 34]. Therefore, in patients with constitutional varus alignment, such as those classified as CPAK type I patients, targeting neutral alignment is difficult, while targeting prearthritic alignment is more feasible and leads to better functional outcomes.

This study had certain limitations. First, although data collection was prospective, the analyses were retrospective. While we performed multivariable regression analyses to adjust for the effects of preoperative demographic information, comorbidities, and alignment, other factors may have influenced our conclusions. Second, our study included only fixed‐bearing medial UKA; therefore, the findings may not apply to mobile‐bearing or lateral UKA. Third, only patients with nonrobot‐assisted UKA were included in this study. Previous study demonstrated that robot‐assisted UKA has a higher rate of reaching the prearthritic alignment [16]. Therefore, a robot‐assisted UKA cohort is needed in the future to further validate the findings of this study.

## CONCLUSION

The findings in this study suggest that personalised alignment strategies that consider the patient's unique anatomical characteristics rather than strictly adhering to neutral alignment may lead to better joint perception and functionality. Specifically, each degree of deviation from prearthritic alignment was associated with a 21% decrease in the likelihood of attaining a forgotten joint. This highlights the importance of considering individual alignment variability during UKA, particularly for patients with constitutional varus alignment.

## AUTHOR CONTRIBUTIONS

Zhaolun Wang designed the study and drafted the manuscript. Wang Deng, Shaoyi Guo and Yong Huang contributed to data collection and analysis. Yixin Zhou provided clinical insights and supervised the project. Yunfeng Zhang contributed to data interpretation and manuscript revision. All authors reviewed and approved the final manuscript.

## CONFLICT OF INTEREST STATEMENT

The authors declare no conflicts of interest.

## ETHICS STATEMENT

The study was approved by the hospital's institutional review board (201909‐06). Informed consent was obtained from all subjects involved in the study.

## Supporting information

Supplementary table 1.

## Data Availability

The datasets generated and analysed during the current study are available from the corresponding author upon reasonable request.
