# Supplementary material for: Restoring prearthritic alignment improves joint perception in medial unicompartmental knee arthroplasty
Source: J Exp Orthop. 2025 Aug 5;12(3):e70389. doi: 10.1002/jeo2.70389 (PMC12322694; doi:10.1002/jeo2.70389)
Supplement: Supplementary file 1 — Supplementary table 1. [file JEO2-12-e70389-s001.docx]

Supplementary table 1. Intra-class correlation coefficient for all measurements.

| Variables | Intra-class correlation coefficient (95%CI) |
| --- | --- |
| Preoperative mHKA | 0.812 (0.750-0.863) |
| mLDFA | 0.823 (0.697-0.892) |
| MPTA | 0.754 (0.551-0.856) |
| Postoperative mHKA | 0.876 (0.808-0.918) |
